# Association between Self-Reported Pain, Cognition, and Neuropathology in Older Adults Admitted to an Outpatient Memory Clinic—A Cross-Sectional Study

**DOI:** 10.3390/brainsci11091156

**Published:** 2021-08-31

**Authors:** Víctor I. Madariaga, Eduard Overdorp, Jurgen A. H. R. Claassen, Inti A. Brazil, Joukje M. Oosterman

**Affiliations:** 1 Donders Institute for Brain, Cognition and Behaviour, Radboud University, 6525GD Nijmegen, The Netherlands; victor.madariagarivera2@donders.ru.nl (V.I.M.); inti.brazil@donders.ru.nl (I.A.B.); 2 Department of Medical Psychology, Gelre Medical Center, 7207AE Zutphen, The Netherlands; e.overdorp@gelre.nl; 3 Department of Geriatric Medicine and Radboud Alzheimer Center, Radboud University Medical Center, 6500HB Nijmegen, The Netherlands; jurgen.claassen@radboudumc.nl

**Keywords:** pain, cognitive dysfunction, dementia, aging, neuropsychology, magnetic resonance imaging

## Abstract

Cognitive impairment has been linked to *reduced* self-reporting of pain. However, it is unclear whether the various cognitive functions are similarly and/or independently associated with such pain report measures. In the present study, we explored how executive functioning (EF), memory, and global cognition relate to self-reported pain and investigated whether underlying neuropathology partially accounts for these results. We used *Lasso* categorical regression to analyze data from 179 individuals visiting a memory clinic. The data included the self-reported pain occurrence, intensity, severity and frequency, clinical diagnoses, neuropsychological scores, white matter hyperintensities, medial temporal lobe atrophy, depressive symptoms, and demographics. Our results showed that *worse* memory and EF performance predicted a *lower* pain occurrence. In those individuals who did report pain, *worse* memory predicted *lower* pain intensity, severity, and frequency levels, but for EF reversed effects were found, with *worse* EF predicting *higher* pain scores. These relationships were only partially explained by reductions in white matter and medial temporal lobe integrity. Similar effects were found for depressive symptoms. Our findings highlight the distinct associations of EF and memory with self-reported pain. A similar pattern of relationships found for both self-reported pain and depressive symptoms may reflect shared latent affective components.

## 1. Introduction

Reliable pain assessment is a major challenge in people with cognitive impairment because the cognitive decline reduces their ability to communicate about their pain. Studies indicate that pain is reported less (i.e., less verbally expressed) by adults with cognitive alterations and that their pain tolerance following electrical stimulation is *increased* [1,2]. However, as recognized by the International Association for the Study of Pain (IASP), impaired communicative abilities may reduce pain self-reporting without altering pain experience itself—in other words, pain experience in persons with cognitive decline may be unaltered, or even *increased*, despite a reduction in self-reported pain [3]. A major problem associated with this discrepancy between the clinical self-reporting of pain and the true experience is that cognitively impaired individuals are at risk of being undertreated for pain [4,5,6]. However, the presence of cognitive impairment is not always associated with a reduction in self-reported pain; the underlying neuropathology may play an important role in how self-reported pain is altered [7]. For example, adults with dementia due to Alzheimer’s disease (AD) and mixed dementia show a *lower* prevalence of self-reported pain compared to individuals with subjective cognitive impairment [1]. In contrast, patients with vascular dementia (VaD) appear to report *higher* pain ratings [8]. This shows that the relationship between self-reported pain and cognitive impairments is still far from being understood.

Part of this problem may be solved by considering how different cognitive functions relate to self-reported, clinical pain. Although it is widely recognized that cognitive functions are crucial for the ability to report pain, it is unclear how self-reported pain is affected by the multiple cognitive dysfunctions seen in e.g., dementia. A previous study found that *worse* global cognitive functioning was associated with *reduced* pain reactivity (i.e., a smaller increase in heart rate after a warning signal of an upcoming painful electrical stimulus, and during the painful stimulation itself) [2]. However, more recent studies have highlighted the importance of a particular set of functions involved in cognitive control (i.e., executive functions; EF) [9,10,11]. Their results suggest that, within the spectrum of cognitive functions, EF may be critical for our ability to reflect upon and report the experienced pain. More specifically, these studies found that *lower* clinical pain ratings were uniquely associated with *worse* EF, both in normal aging [9,10] and in older adults diagnosed with dementia due to AD [11].

However, such findings are at odds with the *higher* self-reported pain levels found in patients with VaD, a disorder typically characterized by pronounced deficits in EF (although large variability exists within the spectrum of VaD) [12]. Thus, impaired EF co-occurs with *higher* rather than *lower* pain ratings in these patients. In line with this, studies on experimental pain sensitivity in normal and pathological aging populations showed that *worse* EF was indeed associated with *higher* verbal and facial expression of pain intensity [13,14]. Taken together, *worse* EF has been associated with *lower* self-reported, clinical pain in both normal aging and patients with AD, but with *higher* pain sensitivity in experimental settings.

One approach to better understand this apparent contradiction is to consider the underlying neuroanatomical substrates. Two studies that examined white matter integrity—a major correlate of EF [15]—in relation to self-reported pain in normal and pathological cognitive aging found that *reduced* white matter integrity (presumably reflecting *worse* EF) was related to *higher* self-reported, clinical pain levels [16,17]. These studies, therefore, corroborate experimental pain studies, in which *worse* EF was related to *higher* pain sensitivity [18,19]; these findings also support the results found in patients with VaD in whom *higher* pain reports and deficits in EF are concurrently present [8]. However, there are no studies that explored the roles of EF and white matter concurrently in self-reported, clinical pain in patients with cognitive impairment. Moreover, only one study examined medial temporal atrophy (MTA)—a major correlate of episodic memory performance [15]—in relation to pain in these populations and reported no association with self-reported pain intensity [17].

The main goal of the present study was to identify unique cognitive predictors of self-reported, clinical pain by analyzing data from persons admitted to an outpatient memory clinic for cognitive complaints. Based on previous studies, we expected an overall association between the performance on tests of EF and the self-reported occurrence (i.e., whether persons reported to be in pain) and level of pain. We explored whether this relationship was positive [11], or if the reversed relationship was present, in which *worse* EF is associated with a *higher* self-reported pain occurrence and *higher* self-reported pain levels [13,19]. We also hypothesized that an *impairment* in memory would be associated with *lower* self-reported occurrence of pain and *lower* self-reported pain levels as shown in another study [20]. Finally, we hypothesized that neuropathological measures such as white matter hyperintensities (WMH) and MTA may explain part of the association between the diverse cognitive functions and self-reported pain [17,21].

## 2. Materials and Methods

### 2.1. Study Design

This cross-sectional, retrospective study was performed using data collected as part of the clinical routine at the memory clinic in Gelre Medical Centre in Zutphen, The Netherlands.

### 2.2. Data Collection and Outcome Measures

Between March 2013 and November 2014, data were obtained during the routine examination from 179 individuals referred to the memory clinic for the assessment of cognitive decline. Their diagnosis followed the criteria established in the fourth version of the Diagnostic and Statistical Manual for Mental Disorders (DSM-IV; American Psychological Association, 1994) via multidisciplinary consensus. Cerebral spinal fluid was analyzed when consensus could not be reached, as stipulated in the Dutch diagnostic guidelines for dementia. Moreover, patients’ data were obtained through general physical and neurological examinations, blood screening, magnetic resonance imaging (MRI), electroencephalography, and neuropsychological tests.

The neuropsychological evaluation included tests of EF, including the Meander’s subtask score from the Amsterdamse Dementie Screeningtest (ADS) [22], the Frontal Assessment Battery (FAB) score [23], and the animals and professions categories test scores for fluency [24]; global cognition (GC), including the 20-item cognitive screening test score [25], and the Mini Mental Status Examination score [26]; and episodic memory (Memory), including the visual association test (VAT) score [27], the eight-word-list test score (immediate recall, recognition and delayed recall from the ADS) [22], and a prose recall test score (immediate and delayed recall) [28].

Several pain variables were measured as well. First, considering the profound cognitive decline (including memory) present in many of the patients (e.g., dementia), we asked them about whether they were in pain at the time of examination and/or in the preceding week (pain occurrence). Scores were recorded on a yes/no scale. When a patient indicated that pain was present, we recorded the following measurements: pain frequency (occasionally, weekly, daily, always); pain intensity, measured by a numerical rating scale (NRS) ranging from zero to ten (zero corresponded to no pain at all and ten to the maximum pain possibly perceived by the individual); pain severity, measured using a 5-point verbal descriptor scale (VDS) ranging from no pain to excruciating pain, and; pain locations, using a drawing of a mannequin. In case a patient was unable to use the mannequin, a healthcare professional indicated the locations based on the patient’s complaints. The locations were coded using six body sites including the head, axial body, and upper and *lower* limbs. Patients were instructed on how to use the pain scales themselves (i.e., explaining the scale itself and the anchors); if patients were unable to use these scales (i.e., they did not understand or follow the instructions), data were not registered for analyses (see the subscript of Table 2 for more information). Although these pain measurements are not currently validated for their use in people with severe cognitive impairment, they are widely used for assessing pain in general (including mild to moderate cognitive impairment) and have shown adequate validity and reliability (except for pain frequency) [29,30,31,32].

Other included data were demographic characteristics, such as sex, age, and education, which was measured using a 7-point ordinal scale, with the following coding system: (1) less than primary education, (2) finished primary school, (3) unfinished low-level secondary education, (4) finished low-level secondary education, (5) finished pre-vocational secondary education or secondary vocational education, (6) finished *higher* professional education or senior general secondary education, and (7) university degree. The clinical diagnosis was registered as well. Additional clinical characteristics were obtained from the patients’ medical records, such as the use of pain medication (peripheral or central mechanism of action, i.e., opioids, pain-related psychotropic and antiepileptic drugs, non-steroidal anti-inflammatory drugs, paracetamol, and other pain medication), the presence of cardiovascular and metabolic diseases (i.e., hypertension, cerebrovascular disease, diabetes mellitus, heart disease), the presence of diagnosed pain disorders (e.g., osteoarthritis), and the 15-point Geriatric Depression Scale score for depressive symptoms (score of six as the *lower* cutoff for depression) [33].

Finally, as part of the diagnostic procedure, MRI was performed with a 1.5 Tesla GE-Signa Horizon LX scanner, using a standardized imaging protocol that consisted of whole-brain axial and coronal fluid-attenuated inversion recovery (FLAIR; TR 10,000 ms, TE 160 ms), sagittal T1 (repetition time TR 300 ms, TE 4 ms), and axial T2-weighted (TR 6500 ms, TE 105 ms) images. The slice thickness for all images was 5 mm with a 2 mm gap. We used the T1-weighted coronal images to rate MTA using the validated Schelten’s scale, a 5-point rating scale (0–4) that is based on the width of the coronal fissure and the temporal horn and the height of the hippocampal formation [22]. T2-weighted axial and FLAIR images were used for the assessment of WMH with the 0–3 Fazekas rating scale [34,35]. All ratings were performed by an experienced geriatrician who has over 20 years of experience in rating MRI’s in an academic memory clinic (>400 new patients/year) and 15 years of experience in rating MRI’s for clinical research (JAHRC), and two experienced neuropsychologists (EO and JMO, who were trained by JAHRC) with approximately 10 years of experience in rating MRI’s for clinical research [15,36]. All raters were blinded to the clinical diagnosis and to the scores from the pain and the neuropsychological assessments.

### 2.3. Data Analysis

Individual neuropsychological test scores were combined in order to obtain domain-specific scores for EF, GC, and memory. We converted test scores into modified z-scores using the overall median, and the median or the mean absolute deviation (MAD/MeanAD) according to the following formula:

$$\frac{\mathrm{Test}\;\mathrm{score}-\mathrm{Median}\;\mathrm{test}\;\mathrm{score}}{\left(\mathrm{MAD}\times 1.486\right)\;\mathrm{OR}\;\left(\mathrm{MeanAD}\times 1.253314\right)}$$

Equation for standardization of values into modified z-scores. The following steps were performed for each variable separately. First, we determined the median score of each individual variable; second, for each patient, we calculated the difference between this median score and their score on this specific variable (the test score). The median absolute deviation (MAD) or the mean absolute deviation (MeanAD) were multiplied by a constant of 1.486 or 1.253314, respectively, for an approximation of the standard deviation. The subtracted score (test minus median test score) was then divided by the MAD or the MeanAD. The latter was used for the eight-word-list delayed recall test score and the Meander’s subtask score from the ADS since the MAD was equal to zero in both cases [37].

After modified z-scores were calculated for each individual test/sub-test, these were averaged across test-sections when they belonged to the same test (e.g., eight-word immediate recall, eight-word recognition, and eight-word delayed recall into the eight-word-list test); and then across tests when different tests belonged to the same domain (e.g., eight-word-list test, VAT, and prose recall test into memory domain) per participant. In case a test score was missing, we used the available scores to calculate the cognitive domains. For all domains (i.e., EF, GC, and memory), a *higher* z-score represented better performance.

Next, we used categorical linear regression (CATREG) combined with optimal scaling and Least absolute shrinkage and selection operator (*Lasso*) regularization [38] and searched for possible predictors of self-reported pain in the whole sample (regardless of the diagnosis). Dependent variables were participants’ self-reported pain occurrence (scaled as nominal), pain intensity by NRS (spline ordinal), pain severity by VDS (spline ordinal), and pain frequency (spline ordinal). Scores for the pain locations were very low; these scores were therefore not further analyzed but only used for descriptive purposes. In Model 1, we included the following predictors (in order): sex (nominal), age (continuous), GDS-15 score (continuous), EF (continuous), GC (continuous), and Memory (continuous); in Model 2, WMH (spline ordinal) and MTA (spline ordinal) were analyzed as predictors together with the cognitive predictors that emerged from Model 1. Using this approach, and following our research question, we could test which cognitive functions uniquely predicted our pain outcomes (when adjusted for age, sex, and GDS-15 score which are possible confounders between such associations in older adults [10,39,40]) (Model 1), and to what extent our target relationships (between cognitive and pain variables) were explained by underlying neuroanatomical changes (Model 2).

An additional model (Model 3) was performed to assess the association between age, sex, EF, GC, Memory, WMH, and MTA as independent variables (in order), and the GDS-15 score as a dependent variable. This last model was executed to analyze if potential relationships between self-reported pain and the cognitive and neuroanatomical predictors (e.g., EF, WMH) were specific to pain, or whether they tapped into a shared underlying affective factor (and should thus result in similar findings for the GDS-15 score compared to those for the pain scores).

*Lasso* regularization was set to a sum of squares of zero as the minimum and one as the maximum, with shrinkage penalties in steps of 0.02 and 0.632 bootstrap resampling, and 5000 iterations. The predictors were identified based on the “optimal model”, which is the model with the lowest expected prediction error and the highest accuracy given the data (for a similar approach see Smeijers et al., 2017 or Brazil et al., 2013 [41,42]).

**Figure 1:**
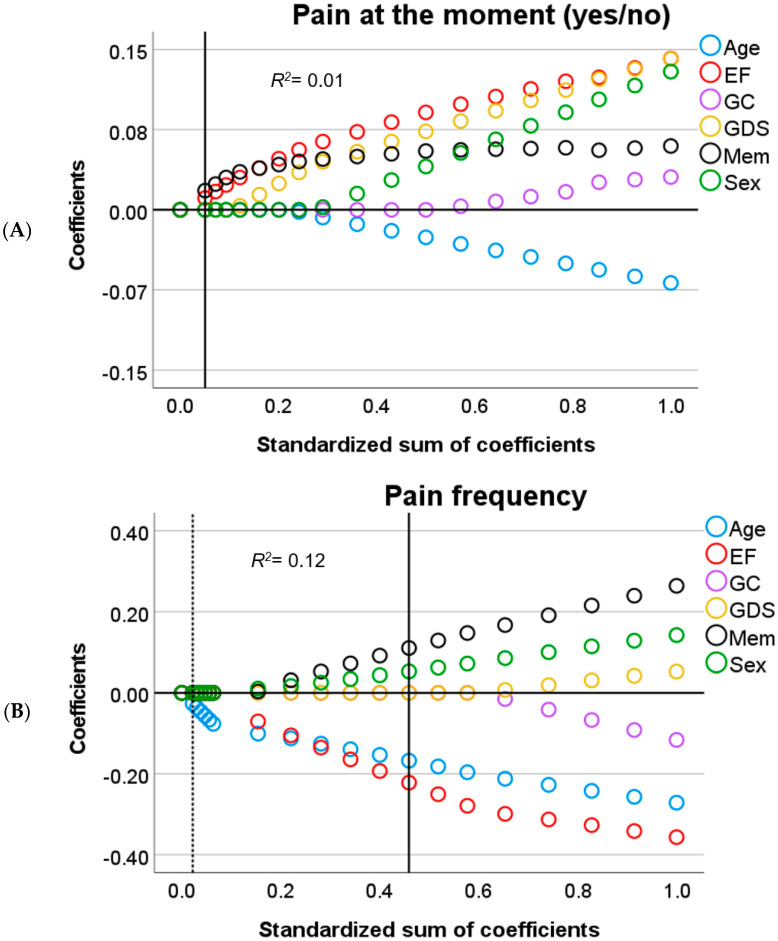
Output charts *Lasso* regression (Model 1). Input predictors are displayed in alphabetical order but were introduced as follows: sex, age, Geriatric Depression Scale score (GDS), executive function (EF), global cognition (GC), and memory (Mem). Identified predictors and the respective dependent variables are: (**A**) EF and Mem as predictors of the occurrence of pain; and (**B**) sex, age, EF, and Mem as predictors of pain frequency. *Lasso* coefficients (colored rings) represent positively and negatively related predictors above and below zero, respectively (y–axis). Vertical lines intersect the coefficients (≠0) from the most parsimonious predictors (dotted line) and the optimal predictors (solid line) as computed in the different models (these lines might coincide). In each subfigure, the values of the x–axis represent the relative influence of all the parameters over the dependent variable before shrinkage (standardized sum of coefficients = 1.0 minus shrinkage = 0, which is equal to 1.0 on the right side). At each stage of shrinkage (increasing from right to left in the x–axis), a *higher* penalty is added until the standardized sum of coefficients is at its lowest (i.e., 0) and shrinkage is at its highest (i.e., 1.0). The smallest prediction error is represented by the solid line tangential to the corresponding shrinkage stage. The predictors with still meaningful associations (≠0) at such a stage are considered optimal predictors with *Lasso* coefficients marked in the y–axis. Regularization-R^2^ is displayed on the top-left corner of each of the plots.

**Figure 2:**
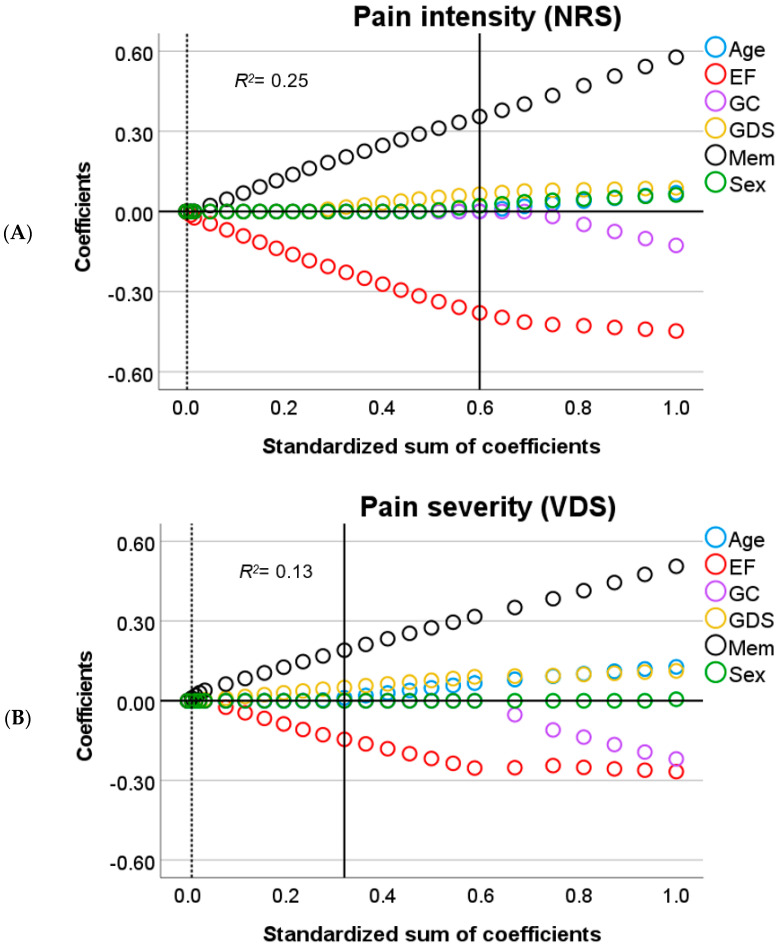
Output charts *Lasso* regression (Model 1)**.** Input predictors are displayed in alphabetical order but were introduced as follows: sex, age, Geriatric Depression Scale score (GDS), executive function (EF), global cognition (GC), and memory (Mem). Identified predictors and the respective dependent variables are: (**A**) sex, age, GDS, EF, and Mem as predictors of pain intensity by NRS; and (**B**) age, GDS, EF, and Mem as predictors of pain severity by VDS. *Lasso* coefficients (colored rings) represent positively and negatively related predictors above and below zero, respectively (y–axis). Vertical lines intersect the coefficients (≠0) from the most parsimonious predictors (dotted line) and the optimal predictors (solid line) as computed in the different models (these lines might coincide). In each subfigure, the values of the x–axis represent the relative influence of all the parameters over the dependent variable before shrinkage (standardized sum of coefficients = 1.0 minus shrinkage = 0, which is equal to 1.0 on the right side). At each stage of shrinkage (increasing from right to left in the x–axis), a *higher* penalty is added until the standardized sum of coefficients is at its lowest (i.e., 0) and shrinkage is at its highest (i.e., 1.0). The smallest prediction error is represented by the solid line tangential to the corresponding shrinkage stage. The predictors with still meaningful associations (≠0) at such a stage are considered optimal predictors with *Lasso* coefficients marked in the y–axis. Regularization-R^2^ is displayed on the top-left corner of each of the plots. Additional abbreviations: NRS= numerical rating scale, VDS = verbal descriptor scale.

**Figure 3:**
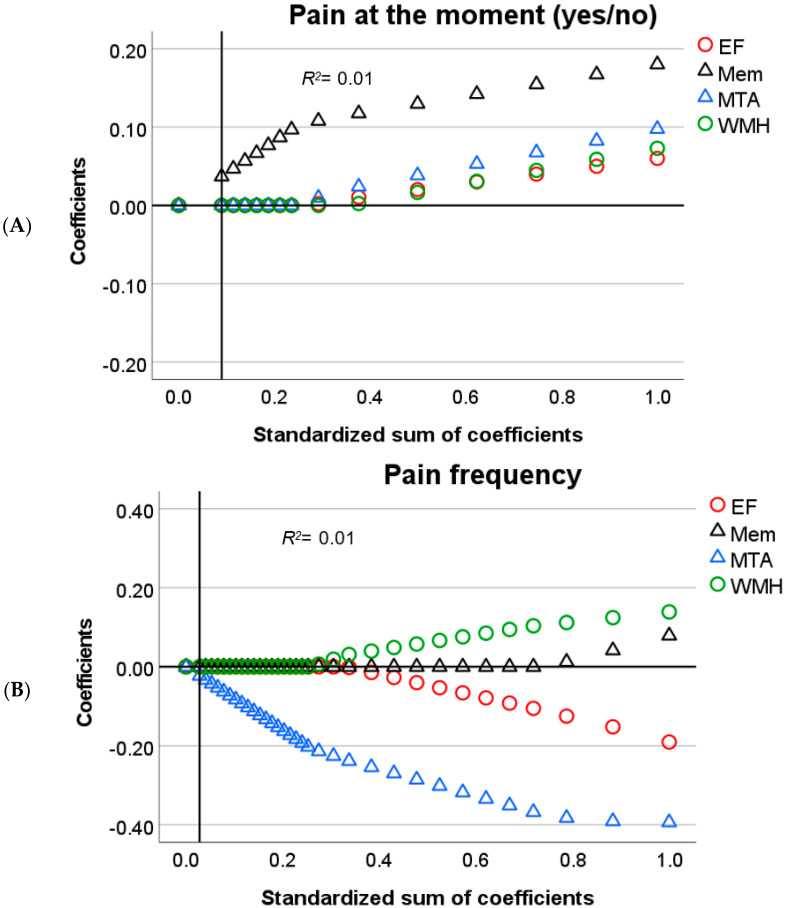
Output charts *Lasso* regression (Model 2). Input predictors are displayed in alphabetical order but were introduced as follows: executive function (EF), memory (Mem), white matter hyperintensities (WMH), and medial temporal atrophy (MTA). Identified predictors and the respective dependent variables are: (**A**) Mem as a predictor of the occurrence of pain; and (**B**) MTA as a predictor of pain frequency. *Lasso* coefficients (colored shapes) represent positively and negatively related predictors above and below zero, respectively (y–axis). Shapes represent variables that in their relations with pain can be visually compared against each other, i.e., the association between pain and EF partially explained by WMH (circles) and the association between pain and memory partially explained by MTA (triangles). These explanatory findings must be visually seen by comparing coefficients against Figure 1. Vertical lines intersect the *Lasso* coefficients (≠0) from the most parsimonious predictors (dotted line) and the optimal predictors (solid line) as computed in the different models (these lines might coincide). In each subfigure, the values of the x–axis represent the relative influence of all the parameters over the dependent variable before shrinkage (standardized sum of coefficients = 1.0 minus shrinkage = 0, which is equal to 1.0 on the right side). At each stage of shrinkage (increasing from right to left in the x–axis), a *higher* penalty is added until the standardized sum of coefficients is at its lowest (i.e., 0) and shrinkage is at its highest (i.e., 1.0). The smallest prediction error is represented by the solid line tangential to the corresponding shrinkage stage. The predictors with still meaningful associations (≠0) at such a stage are considered optimal predictors with *Lasso* coefficients marked in the y–axis. Regularization-R^2^ is displayed on the top-left corner of the plots.

**Figure 4:**
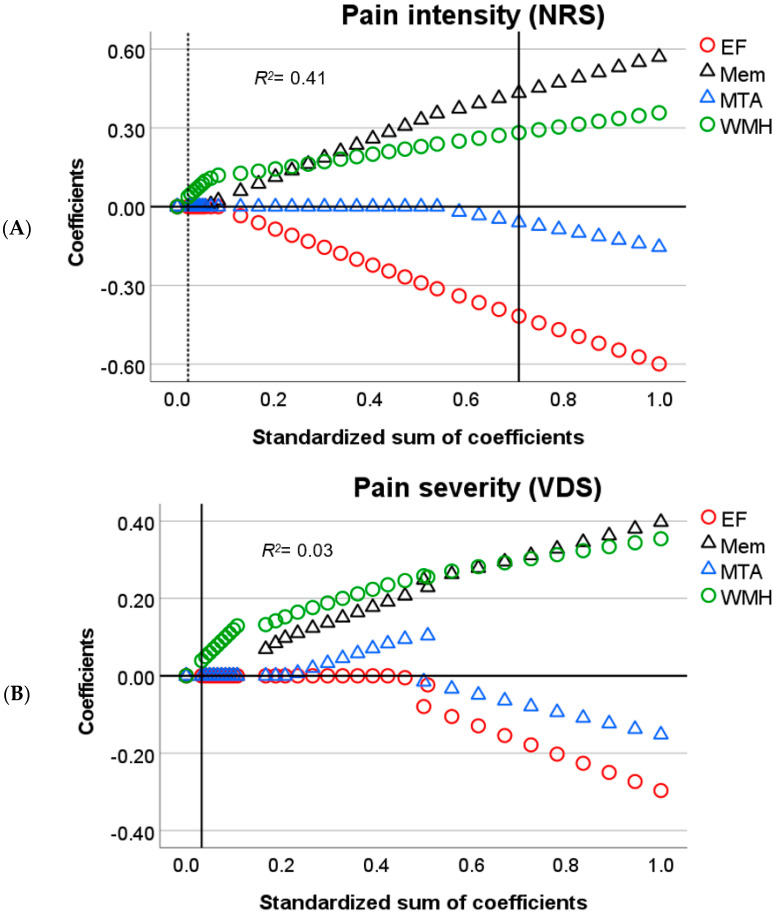
Output charts *Lasso* regression (Model 2). Input predictors are displayed in alphabetical order but were introduced as follows: executive function (EF), memory (Mem), white matter hyperintensities (WMH), and medial temporal atrophy (MTA). Identified predictors and the respective dependent variables are: (**A**) EF, Mem, WMH, and MTA as predictors of pain intensity by NRS; and (**B**) WMH as a predictor of pain severity by VDS. *Lasso* coefficients (colored shapes) represent positively and negatively related predictors above and below zero, respectively (y–axis). Shapes represent variables that in their relations with pain can be visually compared against each other, i.e., the association between pain and EF partially explained by WMH (circles) and the association between pain and memory partially explained by MTA (triangles). These explanatory findings must be visually seen by comparing coefficients against Figure 2. Vertical lines intersect the *Lasso* coefficients (≠0) from the most parsimonious predictors (dotted line) and the optimal predictors (solid line) as computed in the different models (these lines might coincide). In each subfigure, the values of the x–axis represent the relative influence of all the parameters over the dependent variable before shrinkage (standardized sum of coefficients = 1.0 minus shrinkage = 0, which is equal to 1.0 on the right side). At each stage of shrinkage (increasing from right to left in the x–axis), a *higher* penalty is added until the standardized sum of coefficients is at its lowest (i.e., 0) and shrinkage is at its highest (i.e., 1.0). The smallest prediction error is represented by the solid line tangential to the corresponding shrinkage stage. The predictors with still meaningful associations (≠0) at such a stage are considered optimal predictors with *Lasso* coefficients marked in the y–axis. Regularization-R^2^ is displayed on the top-left corner of the plots. Additional abbreviations: NRS = numerical rating scale, VDS = verbal descriptor scale.

**Figure 5:**
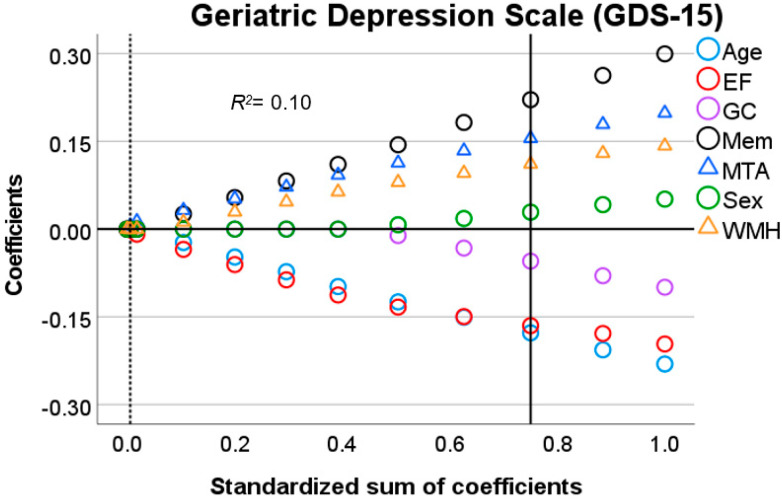
Output chart *Lasso* regression (Model 3). Input predictors are displayed in alphabetical order but were introduced as follows: sex, age, executive function (EF), global cognition (GC), memory (Mem), white matter hyperintensities (WMH), and medial temporal atrophy (MTA). Identified predictors are sex, age, EF, GC, Mem, WMH, and MTA. *Lasso* coefficients (colored rings) represent positively and negatively related predictors above and below zero, respectively (y–axis). Vertical lines intersect the *Lasso* coefficients (≠0) from the most parsimonious predictors (dotted line) and the optimal predictors (solid line) as computed in the model. In each subfigure, the values of the x–axis represent the relative influence of all the parameters over the dependent variable before shrinkage (standardized sum of coefficients = 1.0 minus shrinkage = 0, which is equal to 1.0 on the right side). At each stage of shrinkage (increasing from right to left in the x–axis), a *higher* penalty is added until the standardized sum of coefficients is at its lowest (i.e., 0) and shrinkage is at its highest (i.e., 1.0). The smallest prediction error is represented by the solid line tangential to the corresponding shrinkage stage. The predictors with still meaningful associations (≠0) at such a stage are considered optimal predictors with *Lasso* coefficients marked in the y–axis. Regularization-R^2^ is displayed on the top-left corner of the plot.

In brief, for linear associations, *Lasso* regularization does not only minimize the distance between residuals and the resulting regression line for all parameters (error) but also penalizes overfitting. A penalty is the absolute slope of the associations between parameters and dependent variables, times a constant (lambda), which is added to the error (error + penalty). Lambda represents the weight attributed to the penalty (from 0 to 1). Instead of calculating the best-fitted line that represents the smallest error, *Lasso* regularization minimizes error + penalty. This means that for an error equal to zero, a steep slope and a high lambda in the regression line could result in a *higher* value than for an error *higher* than zero coupled with a less steep slope and low lambda. All possible values for slopes (including zero) and error are evaluated to find the line that minimizes error + penalty given the data. Optimal lambda, on the other hand, is calculated by bootstrapping and selecting the value that yields the least amount of prediction error given the data. The outcome is an equilibrium between prediction error and penalty. The *lower* the penalty, the *lower* the shrinkage of the influence of the predictors over the dependent variable and the *higher* the sum of coefficients (1-shrinkage). Therefore, in Figure 1, Figure 2, Figure 3, Figure 4 and Figure 5, *Lasso* coefficients mainly represent the direction of the association of parameters and dependent variables, but also the slope, at different stages of shrinkage (from penalty = 0 or sum of coefficients = 1 to penalty = 1 or sum of coefficients = 0). The prediction error is represented by vertical lines that show at which shrinkage stages prediction errors are the smallest, i.e., the optimal model. The smallest number of predictors that can explain the dependent variable is represented by a second vertical line, i.e., the most parsimonious model (see the subscript of Figure 1, Figure 2, Figure 3, Figure 4 and Figure 5 for a graphical representation) [43].

Moreover, we wanted to explore whether our findings were driven by specific predictors rather than by clinical diagnoses, e.g., that a unique association between pain intensity and memory was driven by memory predictors rather than by an underlying diagnosis characterized by memory deficits (i.e., single- or multiple-domain amnestic mild cognitive impairment (aMCI) and/or AD). To do this, we compared the pain outcomes between four diagnostic categories: subjective cognitive impairment (SCI), aMCI, dementia related to AD, and other cognitive impairments (OCI) (e.g., Parkinson’s disease, vascular dementia, mixed dementia, parkinsonism, dementia, frontotemporal dementia, among others). Data from the whole sample were examined for normality by visually inspecting the histogram and Q-Q plots and using a skewness threshold of |1.0| as maximum. Potential group differences were analyzed using the Kruskal-Wallis test for ordinal and continuous observations and Pearson’s Chi^2^-test for frequencies; a *p*-value below 0.05 was used to determine statistical significance. Data with missing values (i.e., not registered during the clinical examinations) were excluded from the analyses.

Finally, we tested whether cognitive variables were associated with WMH and MTA in our sample using Spearman’s correlation analyses.

All statistical analyses were executed using the IBM SPSS package for statistics, version 27.0 (IBM Corp, Armonk, NY, USA).

## 3. Results

### 3.1. Demographics and General Health

A detailed characterization of the studied population is presented in Table 1. In brief, the whole sample encompassed 179 individuals with a median age of 78 (IQR = 12), and more than half of them were women. More than half of the study population had a low educational level (i.e., ranging from less than primary education to finished low-level secondary vocational education) and almost half of them had at least one comorbidity reported in the medical records; hypertension was the most frequent. Concerning pain history, around 40% of the participants had a known painful condition, but only around one-third used pain medication (as reported in the medical records; for more information, see the subscript of Table 1). Additionally, a majority of individuals had some level of depressive symptoms, but only a few could be categorized with depression (GDS-15 score of six or more [33]). Finally, the most prevalent diagnoses were AD, aMCI, and SCI, which comprised two-thirds of all diagnoses. For the remaining 52 patients, diagnoses were diverse and included conditions such as vascular dementia, frontotemporal dementia, parkinsonism, and Parkinson’s disease, among others (see Table 1).

### 3.2. Predictors of Pain in Cognitive Decline 

To simplify the analysis of predictors, we will only discuss significant findings with respect to the relationship between self-reported pain and the cognitive variables (Figure 1 and Figure 2). Next, we focus on the effect of neuroanatomical correlates that could explain such a relationship, in accordance with our research question (Figure 3 and Figure 4).

The outcome model from the first *Lasso* regression (Model 1) showed that EF and memory are relevant predictors of self-reported pain occurrence (Figure 1A). The pattern of results suggests that *worse* memory and EF relate to *lower* self-reported pain occurrence. After including WMH and MTA (Model 2), memory remained the only relevant predictor (Figure 3A).

In patients who did report that they were experiencing pain (i.e., who reported pain occurrence), several predictors were associated with the characteristics of this pain (i.e., intensity, severity, frequency). For pain frequency, the first model showed a negative relation with EF and a positive relation with memory (Figure 1B). Here, *lower* memory performance predicted *lower* pain frequency scores, whereas for EF opposite results were found, with *lower* EF predicting *higher* pain frequency. In the second model, MTA was the only associated predictor, with *higher* atrophy rates relating to *lower* frequency reports (Figure 3B).

For the pain intensity (NRS) scores, the results were similar to those for pain frequency, showing that *worse* memory predicted *lower* pain intensity, whereas *worse* EF predicted *higher* pain intensity scores (Figure 2A). When WMH and MTA were added to the model, the effects of EF and memory were still present, but with additional effects from WMH and MTA (Figure 4A). For WMH, we found that more white matter damage related to *higher* pain intensity scores, whereas MTA showed an opposite effect, with *higher* atrophy rates predicting *lower* pain intensity scores.

Regarding pain severity (VDS scores), the first model showed a negative relation with EF and a positive relation with memory, similar to the results found for the pain frequency and intensity scores (Figure 2B). However, in the second model, WMH was the only selected predictor (Figure 4B) showing that an increase in white matter damage predicted *higher* pain severity scores.

Taken together, these results suggest that for pain frequency, intensity and severity, *worse* EF relates to *higher* pain scores, whereas *worse* memory relates to *lower* pain scores. These results were only partially accounted for by changes in white matter and medial temporal lobe integrity.

### 3.3. Predictors of Depression-Related Complaints

We repeated the analysis for depressive symptoms to examine whether the observed associations of neuropsychological and neuroanatomical correlates with self-reported pain reflect a pain-specific association, or whether they are merely indicative of a change in affective processing in general. This analysis showed a positive relation of depressive symptoms with memory, WMH, and MTA, and a negative relation with GC and EF (Figure 3). These findings slightly contrast the results found for the NRS and VDS scores, since this time, an effect of GC was found. Moreover, a reversed association with MTA emerged (positive for depressive symptoms, negative for pain complaints). The associations between EF, memory, and WMH with GDS were similar to those found for NRS and VDS scores.

### 3.4. Pain Profile of Patients at Memory Clinic

A description of the self-reported pain in the sample categorized by the diagnostic group is presented in Table 2. Most patients were able to complete the pain scales, although some missing data were present. This included data of: four patients for the NRS, nine patients for the VDS, one patient for pain frequency, and three patients for pain locations. No significant group differences were found for any of the pain outcomes.

### 3.5. Relation between Cognitive and Neuropathological Variables

To assess whether cognitive variables were indeed associated with neuropathological changes in our sample, we performed non-parametric correlation analyses. WMH was negatively associated with EF (ρ = −0.35, *p* < 0.001), GC (ρ = −0.25, *p* < 0.01), and memory (ρ = −0.21, *p* < 0.01). MTA was negatively associated with EF (ρ = −0.36, *p* < 0.001), GC (ρ = −0.38, *p* < 0.001), and memory (ρ = −0.36, *p* < 0.001). These correlations show that an increase in white matter damage and medial temporal lobe atrophy relates to *worse* cognitive performance.

## 4. Discussion

The goal of the present study was to elucidate how different cognitive functions relate to self-reported pain in older adults visiting a memory clinic. To better understand these associations, we tested whether underlying damage of the white matter and atrophy of the medial temporal lobes could partially account for these results. Our main findings are that both memory and EF are uniquely associated with all the subjective pain measures even when adjusted for sex, age, and GDS-15 scores as possible confounders between pain and cognition. First, we found that both *worse* memory and EF predicted *lower* self-reported occurrence of pain. Second, our findings showed that *worse* memory functioning was related to *lower* self-reported pain intensity, severity, and frequency. In contrast, opposite effects were found for EF, with *worse* EF being associated with *higher* self-reported pain intensity, severity, and frequency reports. Our results also showed similar effects of the corresponding neuropathological measures, with *more* white matter damage (suggestive of *worse* EF) relating to *higher* self-reported pain intensity and severity, and *more* MTA (suggestive of *worse* memory) related to *lower* self-reported pain intensity and frequency. However, EF and memory remained unique correlates of pain intensity, and memory still predicted the self-reported occurrence of pain, after adding the neuroanatomical measures to the analyses. Therefore, we propose that memory and EF are important independent predictors of self-reported pain in older adults visiting the memory clinic and that these effects can only be partially accounted for by an underlying reduction in white matter and medial temporal lobe integrity.

An intriguing question is how to interpret the findings of this study; how can we explain the observed unique roles of memory and EF in self-reported pain? The finding that a *lower* reported pain occurrence related to both *worse* memory and *worse* EF is in line with previous studies showing a general decline in pain self-reporting as cognition becomes more impaired [1,2]. Therefore, this relationship may not be specific for the cognitive domains of memory and EF; it may merely reflect the decrease in capability to report pain as the severity of cognitive impairment increases. This explanation may also apply to the relationship in which *worse* memory was associated with *lower* self-reported pain intensity, severity, and frequency. However, our results contrast previous studies in which such an association between pain and memory functioning could not be confirmed [9,10,11]. One potential explanation for this is the high prevalence of clinical conditions characterized by memory deficits in our study, namely patients with aMCI and with dementia due to AD. This may have resulted in that memory deficits overrepresented the level of cognitive impairment and, therefore, memory emerged as a strong and independent predictor of our pain outcome measures. Nonetheless, we cannot rule out that memory has a unique role in self-reported pain, as it has been shown to be involved in the precision for discrimination of intensities and duration of painful stimuli [44]. Moreover, memory loss is strongly related to the level of MTA as shown in both our results and the literature [45,46] and, despite the small association between this marker of atrophy and pain, our results suggest a partial interrelation between these three factors. We also compared the pain outcomes among the different clinical diagnoses (differing in the level and locus of cognitive impairment), but we did not find any significant effects. This suggests that the influence of memory may not merely represent an underlying effect of the severity of cognitive impairment or diagnostic entity, but it may reflect a memory-specific association. Taken together, these findings suggest that memory may play a unique role in our ability to indicate the presence, intensity, and frequency of pain. Nonetheless, because of the correlational nature of our data, the interpretations remain speculative and should be replicated in future studies, focusing on different clinical populations as well.

In relation to EF, our findings are in line with experimental studies that demonstrated that *worse* EF is associated with *higher* self-reported pain intensity [13,14,18,47]. In this context, studies have revealed different mechanisms by which EF could influence pain. For instance, one study showed that cognitive inhibition explained 20% of the pain response variance in an experimental pain paradigm in pain-free older individuals. In fact, this study showed that a decreased cognitive inhibition and shifting ability were related to *higher* facial responses due to pressure pain [19]. This could reflect the involvement of EF in endogenous pain inhibition [48]. Such a control mechanism can be supported by our results in which an *increased* level of WMH was related to both *worse* EF and *higher* self-reported pain levels; white matter forms the connecting pathways between brain regions and, in this sense, is of crucial importance for top-down pain modulation. This is consistent with other studies that showed a positive relationship between WMH and self-reported pain intensity and/or effect as well [16,17]. To what extent this relationship represents a pain-specific inhibitory mechanism is unclear. An alternative explanation is that this reflects a more general change in affective regulation which results in *higher* pain ratings. If this is indeed the case, the effect of EF would also be detected in non-pain-related affective domains. This is a reasonable explanation, as the prefrontal cortex—which is considered to be the major neuroanatomical region responsible for EF—is involved in emotion regulation, cognitive control, and working memory, among other functions [49,50]. To test this hypothesis, we repeated the analysis for depressive symptoms and found similar associations with EF, memory, and WMH. This suggests that in our population, part of the effect of cognition and neuropathology on pain could be related to general effective processing.

This similarity between our results and those found in experimental studies is remarkable given that these designs (experimental and observational) do not always converge on the same conclusions. Experimental research attempts to control all the possible confounders and find specific causal relationships [51]. In contrast, observational studies, especially those performed utilizing retrospectively collected data or cross-sectional designs, have little control over the exposure or the data collection itself [52]. Nevertheless, such a convergence allows us to infer about the further generalizability of experimental paradigms, for example, whether this applies to those cases where experimenting is not advised (e.g., ethical considerations for pain research in severe cognitive impairment [53]).

Since our study design does not allow us to draw causal conclusions, an alternative explanation of our results is that pain influences cognition instead of, or in addition to, the opposite association (i.e., cognition influences pain expression). In general, it is known that cognition and pain have a reciprocal influence, in which pain negatively affects cognitive functions and in which cognitive states can increase or decrease pain [48]. In line with this, studies show that cognitive functioning is *reduced* in the context of (acute and chronic) pain [54,55,56], and more specifically, studies indicate that EF is one of the cognitive domains that is often impaired in patients with chronic pain [57]. Therefore, the results of the current study could also be suggesting negative effects of pain on EF and underlying white matter integrity [54,58,59]. Although this is a likely explanation, it cannot account for the findings in which *lower* self-reported pain levels were associated with *worse* memory functioning and *more* medial temporal lobe atrophy. 

This study has several strengths. As far as we are aware, this is the first study that concurrently examines diverse cognitive functions and the underlying neuropathological correlates in relation to self-reported pain in older adults visiting a memory clinic. By considering both predictors together, we were able to demonstrate unique contributions of EF and memory which can be partially explained by the corresponding underlying neuroanatomical measures. Another strength is that this is the first article that utilizes a state-of-the-art non-parametric statistical approach for the selection of pain predictors in cognitive impairment, i.e., the *Lasso* categorical regression. This tool allows for the definition of each type of variable in the same model to obtain predictors that yield the least amount of error. Despite these strengths, several potential limitations need to be discussed. First, pain-related subjective data were obtained from individuals with different levels of cognitive impairment in a clinical setting which limits the reliability of data collection (e.g., particularly for the VDS there were some missing data, as not all patients comprehended this scale; also, in case of more severe cognitive impairment, drawing of the pain locations on the mannequin were mostly performed by the testing assistant). Second, due to the retrospective nature of the data, the pain medication and the comorbidity variables could not be confirmed by, e.g., a relative of the patient. Data for these variables may thereby have been incomplete. Third, as stated above, the cross-sectional design of this study does not allow for causal analysis of the associations between cognition, neuroanatomy, and pain. Fourth, the natural frequency of the diagnostic subgroups and the sample size did not allow for further analysis within each diagnostic group. In line with this, since we used a convenience sample of older adults visiting the memory clinic, generalizability is limited. Moreover, a high number of individuals were diagnosed with AD, whereas other types of cognitive impairment were less represented. Fifth, WMH were rated in the entire cerebrum, without distinguishing between the different lobes; we, therefore, do not know to what extent white matter damage involved the prefrontal cortex or connecting (sub)cortical brain regions that play a role in EF. Therefore, further studies are needed to examine neurocognitive correlates of pain self-reporting in more detail in a sufficiently powered and balanced sample and to explore whether the current findings are applicable to different populations.

Our study has several important clinical implications. First, this study highlights the importance of using alternative pain assessment methods in patients with cognitive impairment, as we clearly demonstrate that cognitive functions play a crucial role in the self-reported occurrence, intensity, severity, and frequency of pain. Although this is a well-known recommendation [60,61,62,63,64], our study underscores the relevance of such considerations in the case of memory impairment, especially because here, pain may be present more frequently and more intensely than is being reported. Second, if executive functioning is impaired, an *increase* in pain experience may be expected, although it is currently unclear whether this may be specifically associated with pain or with affective processing in general. Finally, neuropathology itself can also be regarded as an independent marker of the self-reported pain, with white matter damage relating to *higher* pain ratings and medial temporal lobe atrophy relating to *lower* pain ratings and frequencies.

## 5. Conclusions

To conclude, in line with the current literature regarding neurocognitive correlates of self-reported pain in older adults, our results confirmed that memory and executive functioning are independently associated with self-reported pain. Worse memory was related to *lower* self-reported occurrence, intensity, severity, and frequency of pain. Lower executive functioning was also related to *lower* self-reported pain occurrence but was related to *higher* pain intensity, severity, and frequency instead. Additionally, our results suggest that these relationships are partially explained by white matter and medial temporal lobe integrity. In light of the limitations of this study, no causal interpretations could be drawn on whether this is an actual effect of cognition on self-reported pain. However, our results highlight the importance of considering comprehensive pain assessment methods in older individuals with cognitive impairment—especially because verbal pain expression may be strongly influenced by their lack of ability to communicate it and reflect on their own experience.

## Figures and Tables

**Table 1:** Characteristics of the study population.

Characteristics	Value
*Demographics*	
N	179 (100%)
Women	97 (54.2%)
Age	78 (IQR 12)
Low educational level	104 (58.1%)
*Comorbidities*	
Hypertension	88 (49.2%)
Cerebrovascular disease	37 (20.7%)
Diabetes Mellitus	29 (16.2%)
Heart disease	57 (31.8%)
*Pain history*	
Painful condition	73 (40.8%)
Pain medication	60 (33.5%)
*GDS-15 (n = 172)*	
Depressive symptoms	2.0 (IQR 2.0)
Depression (score ≥ 6)	15 (8.8%)
*Cognitive diagnoses*	
Alzheimer’s disease	60 (33.5%)
Amnestic mild cognitive impairment	45 (25.1%)
Subjective cognitive impairment	22 (12.3%)
Other cognitive impairments:	52 (29.1%)
Mixed dementia	8 (4.5%)
Dementia	4 (2.2%)
Parkinsonism	4 (2.2%)
Stroke	4 (2.2%)
Vascular dementia	4 (2.2%)
Delirium	3 (1.7%)
Frontotemporal dementia	3 (1.7%)
Deferred diagnosis	11 (6.1%)
Others (~1% each)	11 (6.1%)

Mixed dementia represents the combined pathology of Alzheimer’s disease and vascular dementia. Others: Cerebral amyloid angiopathy (*n* = 2), Lewy Body Dementia (*n* = 1), normal pressure hydrocephalus (*n* = 1), Parkinson’s disease (*n* = 2), Obstructive sleep apnoea syndrome (*n* = 1), progressive aphasia (*n* = 1), Space occupying lesion (*n* = 2), Vascular Encephalopathy (*n* = 1). Low educational level includes the levels from less than primary education to finished low-level secondary education. Pain medication: medical records reported current use of at least one opioid drug in *n* = 15 (8.4%); at least one pain-related psychotropic or antiepileptic drug in *n* = 15 (8.4%); at least one anti-inflammatory drug or paracetamol in *n* = 40 (22.3%); and other pain medication in *n* = 2 (1.1%). Values are represented as absolute quantity and percentage of the total, except for age and depressive symptoms which are represented as the median and interquartile range (IQR). Abbreviations: GDS = Geriatric Depression Scale.

**Table 2:** Pain occurrence in the sample of patients categorized by diagnosis in groups of subjective cognitive impairment, amnestic mild cognitive impairment, dementia related to Alzheimer’s disease, and other cognitive impairments, and pain characteristics in the subsample of patients who self-reported pain.

Pain	SCI	aMCI	AD	OCI	Statistical Tests
Pain occurrence *n* (%)	11 (50.0)	15 (33.0)	19 (32.0)	24 (46.0)	X^2^ = 4.20, *p* = 0.24
Pain intensity NRS (0–10) Median (IQR)	6.0 (4.0)	6.0 (2.6)	5.5 (4.3)	6.8 (3.1)	H = 2.53, *p* = 0.47
Pain severityVDS (0–4) Median (IQR)	1.0 (1.3)	1.0 (1.0)	1.0 (2.0)	2.0 (2.0)	H = 1.59, *p* = 0.66
Pain frequency (1–4) Median (IQR)	3.0 (2.0)	1.0 (2.0)	3.0 (2.0)	3.0 (2.8)	H = 3.93, *p* = 0.27
Pain locations (1–6) Median (IQR)	1.0 (2.0)	1.0 (0.3)	1.0 (0.5)	1.0 (1.0)	H = 2.67, *p* = 0.45

AD = Dementia related to Alzheimer’s disease, IQR = interquartile range, aMCI = amnestic mild cognitive impairment, NRS = numerical rating scale, OCI = other cognitive impairments, SCI = subjective cognitive impairment, VDS = verbal descriptor scale. Kruskal-Wallis’s test (H) and Chi^2^-test (X^2^). *p* < 0.05 as statistically significant. Pain occurrence was self-reported by *n* = 69 older adults; pain intensity (*n* = 65) was scored from 0 to 10; pain severity (*n* = 60) was scored from no pain to excruciating pain; pain frequency (*n* = 68) was scored as occasionally, weekly, daily, always; and pain locations (*n* = 66) was scored by the number of locations grouped as axial, left upper-limb, right-upper limb, head, and neck, left-*lower* limb, right *lower* limb.

## Data Availability

Data and analysis outputs are available upon request to the corresponding author.
